# Supplementary material for: Mixed-precision weights network for field-programmable gate array
Source: PLoS One. 2021 May 10;16(5):e0251329. doi: 10.1371/journal.pone.0251329 (PMC8109814; doi:10.1371/journal.pone.0251329)
Supplement: Attachment — Submitted filename: reply_letter.pdf [file pone.0251329.s001.pdf]

Revision Notes

(To be attached to a revised manuscript Reference No. PONE-D-20-36981R1)

March 3, 2021.

Dear Dr. Emily Chenette,
Editor-in-Chief,
PLOS ONE

Dear Reviewers,

Thank you very much for your kind consideration and careful evaluation of our following manuscript.

Reference No.: PONE-D-20-36981R1
Title: Mixed-precision weights network for field-programmable gate array implementation
Authors: Ninnart Fuengfusin and Hakaru Tamukoh

We have revised our paper in line with the suggestions made by the associate editor and reviewers. We are extremely grateful for the thorough review and useful suggestions. We have incorporated these suggestions in the revised manuscript, which we feel has improved the quality and understandability of the paper.

We would like to address submission issues in our previous submission as follows. We reattached a pdf file as "manuscript" and a tex file as "other". We also added a reference to Table 6 in the text.

We would like to inform that all number of page, reference, listing, and table in this letter referred to the Revised Manuscript with Track Changes.

We would like to resubmit our paper for publication in the PLOS ONE.
Thank you very much for your efforts and time.

Best regards,

Ninnart Fuengfusin
Kyushu Institute of Technology, Japan
fuengfusin.ninnart553@mail.kyutech.jp

Reply to reviewer 1:

Comment 1:

A comprehensive analysis, especially on larger and more complex networks such as the ResNet family, would be needed to provide a better understanding of the true value of the proposed scheme.

Reply 1:

We conducted an additional experiment with the ResNet-18 model and CIFAR10 dataset in the CIFAR10 section (page 16-17). Using results from 70 rounds of Bayesian optimization (BO) search, we did a comprehension analysis on outcome combinations of mixed-precision weight network (MPWN). We found out that there are patterns of heuristic rules appeared in the top-5 combinations as shown in Table 6 (page 17).

Comment 2:

For example, the authors devote a long discussion on "XNOR signed-bit", which involves multiplying a floating-point activation with a binary weight. At the heart of this multiplication, essentially it is a simple implementation of changing the sign of a value by flipping the MSB bit of a floating-point number depending on the multiplied binary weight. A smarter synthesis tools could have easily performed that operation when instructed to multiply by -1. Therefore, the significance of the long discussion on how bypassing current limitation of one specific high-level synthesis tools is very limited.

Reply 2:

We agree that by multiplying an input feature with a constant weight -1, the synthesis tool should only toggle only the signed-bit of the input feature. However, since this binary weight is a variable that can be in {0, -1}, this task becomes more complicated. With two possible states of *int1* (1-bit integer), the smarter compiler may construct the XOR-signed bit (XSB) if it understands both binary representations of 16-bit floating point (*half*) and *int1*. The complier must also detect that only the signed-bit is changed in this multiplication (which may be weird from the multiplication with 0). Maybe there is the smart compiler that achieves both of these considerations, however, this task is not as simple as multiply by -1.

Comment 3:

I suggest the authors to focus either on the optimization algorithms with more comprehensive network structures and datasets, or the authors may focus on the hardware implementations of different combination of network layers and show how they would have made the design more efficient than a fixed-precision network.

Reply 3:

Thank you for your suggestions, we revised with both your suggestions. We added the CIFAR10 section (page 16-17) that we discussed in **Reply 1**, and we also have revised our FPGA implementation of **FBTBF** to **FTTTF** (a better combination to **FTTTT** in our revised ASB) instead. Even though we did not implement the **T** layer with the sparse matrix multiplication or convolutional operation, we implemented **T** layer with a ternary bitwise operation (TBO) [30] instead. We discussed the TBO thoroughly in the ternary bitwise

operation section (page 10-11). Using the same concept as XSB, the TBO designed for the ternary weight {-1, 0, 1} using only XOR and AND gates. As shown in Table 7 (page 18), TBO consumes only 0 latency and 32 FFs while the 32-bit floating point multiplication consumes 1 latency, 3 DSP48E, 130 FFs, and 150 LUTs.

Reply to reviewer 2

Comment 1:

The article presenta a nice work of using mixed-precision weights networs (MPWN) to implement a quantization neral network. The approach is well described, as well the software simulation and hardware implementation.

Reply 1:

Thank you for your kind response.

Reply to reviewer 3

Comment 1:

The authors need to compare their results with other works in the literature. There are many other compression approaches based on quantization applied to the Deep Neural Networks in the literature. Which does the advantages of the proposal presented in this manuscript?

Reply 1:

We provided the literature of the mixed-precision model in the last paragraph on page 2. Our main advantages compared with previous methods are we thoroughly explored the ASB metric with all possible combinations of the LeNet-5 model. Using this insight, we able to correct a negative correlation between the test accuracy and ASB using the min-max normalization and weighted average with ASB.

In terms of hardware implementation, we included a comparison with a prior work, Rongshi et al [39]. in Table 10 (page 20), 11 (page 21), and 12 (page 21). Rongshi et al. implemented a 32-bit floating-point LeNet-5 model on Xilinx Zybo Z7 board with Vivado HLS 2018.2. Our model performed better in terms of latency by 11.62 times with a trade-off with more hardware resources.

Comment 2: The authors used a High-Level Synthesis (HLS) tool to implement the proposal on FPGA. However, these kinds of tools insert a high overhead resource in the generated hardware. The authors must explain more about that. Does the HLS decrease the results performance? Does it not better to implement without HLS?

Reply 2:

We have added the explanation related HLS and HDL in the second paragraph on page 3 as follows:

"This allows users to rapidly develop applications with an interface of a C-like language with fewer constraints from the HDL language. With this property, HLS is especially useful when applies with the deep learning algorithm that its state-of-the-art algorithm has been being rapidly changed. However, a drawback of HLS compared to optimized handcrafted HDL is that HLS-generated HDL code may cause a higher latency and hardware utilization [21]. For instance, Ordaz *et al.* [21] compares HDL and HLS

implementations of cyclic redundancy check (CRC). Ordaz *et al.* found out that HDL implementation consumes less than LUT by 1.58 times and less amount of latency by 2.63 times."

Reply to reviewer 4

Comment 1.

There are a number of papers in computer architecture and FPGA conferences as well as in ML/DL conferences/journals that deal with mixed precision networks. However, the current paper has no discussion of such prior work. Hence, it does not set the background for the current work properly. There is no comparison, qualitative and quantitative with such prior works either which is important to see where lies novelty in the current paper.

Reply 1:

We provided literatures of the mixed-precision model in the last paragraph on page 2. In this paragraph, to the best of our knowledge, we discussed our novelties comparing with the previous works as follows:

1. We utilized Bayesian optimization to search a suitable weight space instead of using the reinforcement learning or differentiable architecture search [20].
2. We also provide ASB scores that we specifically designed for the MPWN model.
3. We included a sparsity as a part of ASB and we left a choice to not quantization into the search space.

Comment 2.

The motivation for using binary, ternary and FP weights in a neural network is not clear. It seems that the authors have based their decision based on their prior work [19]. However, the heuristics based prior work [19] does not explain how "large" or "small" is to be determined in the context of the current paper.

Reply 2:

We have added definitions of "large" and "small" in the last paragraph of page 7 as follows:

"In this case, we define a large number as a number that more than one positive standard deviation from a mean and we define a small number as a number that less than or equal to one positive standard deviation from the mean.

Comment 3:

ASB is defined to be = (a+s+bnor)/3. However, the three quantities are not dimensionally equivalent. For instance, how is sparsity defined as a number - that should be explained and how dimensional analysis considerations are taken into account in this formula should be explained.

Reply 3:

We have added a comprehension analysis on ASB from the second to the last paragraph of page 14. We found out that are differences in distributions between *a, s,* and *b_nor*. Each one of them does not contain a same range or variance. *a* contains especially low variance comparing with other two metrics due to a small range between minimum and maximum values of *a*. This causes the negative Pearson correlation between *a* and ASB. Due to this problem, we did some preprocessing and modifications to ASB as follows:

1. We rescaled each metric of ASB to a same range of [0, 1] by using a min-max normalization.

2. Using the min-max normalization requires actual minimum and maximum values of the distribution. However, this is not feasible without training all possible combinations of MPWN. Therefore, we proposed estimating rules to find estimated maximum and minimum values from the full precision, ternary and binary models instead.

3. To further balance the correlation between each parameter of ASB, we proposed to use ASB with a weighted average with weights: *alpha, beta,* and *gamma*.

By using a min-max normalization, we able to improve the Pearson correlation between *a* and ASB from the negative correlation -0.2249 to the positive correlation 0.2816.

Comment 4:

FPGA implementation described in this paper is regular textbook implementation. Unfortunately, the reviewer failed to see any novelty related to FPGA implementation.

Reply 4:

Our hardware novelty is mainly contributed from the XOR-signed bit (XSB). Using the same concept of XSB, we succeeded to implement a ternary bit-wise operation (TBO) [30] which we discussed in the Ternary bitwise operation section on page 10 and 11. Other than this is proof of work that our MPWN model can be implemented using both XSB and TBO.

Comment 5:

The motivation for XNOR Signed Bits is unclear. The authors claim that it is for multiplying float with binary values (-1, 1). However, in IEEE float representation, the sign bit is implicit and it only needs to be changed from 0 to 1 or vice-versa. Detecting whether binary weight is -1 or 1 and simply flipping the 32nd or the 64th bit in float representation would change the sign. Why is the algorithm in Listing 1 needed? Also, Vivado HLS supports bit-accurate data type. Conversion between datatypes are not needed.

Reply 5:

We have added a discussion on in the first paragraph of page 18. In terms of behavior, this should operate in a same manner as XSB. However, by "Detecting", this requires a control logic which is expensive in Vivado HLS. We did a comparison between XSB and Listing 3 (page 18) that utilizes the control logic by using C synthesis with Vivado HLS 2019.2. We found out that the Listing 3 consumes 2 cycles at 100 MHz, 2 DSPEs, 99 FFs, and 150 LUTs, while XSB consumes only 0 cycles (or in real-world 1 cycle) and 2 FFs.

Comment 6:

The section on experiments and results is limited to the authors' implementation in software and FPGA. This is a major concern with this paper as it makes no comparison with prior work. It is well known that FPGA implementations are typically targeted for hardware acceleration and hence they are supposed to be faster than CPU only implementation.

Reply 6:

In terms of the software, we did compare with both BinaryConnect (BC) [9] and Ternary weight networks (TWN) [11] without a scaling factor in Table 3 (page 14). In terms of hardware, we have added a comparison with prior work, Rongshi et al [39]. in the Table 10 (page 20), 11 (page 21), and 12 (page 21).

Comment 7:

. In Table 5, the latency of multiplication between half and int1 cannot be 0. There will be some delay associated with this operation. The absolute value of the delay can be less than 10 ns (100m MHz clock used in the design) and hence if registered inputs go to XSB and outputs of XSB operation are registered, the latency will be 1 clock cycle. Even if inputs and output are not registered, the absolute delay is there. Writing latency as 0 is not the correct way to interpret this delay.Half is 16 bits in representation. If one multiplies it with int1 i.e. 1 bit integer,the result is still 16 bits. It is not clear this resulted in only two FFs because a single FF can only store 1 bit.

Reply 7:

We only provided the latency that was reported from C synthesis with Vivado HLS. However, as you mentioned, the real latency cannot be 0, therefore we have added the following statement in the first paragraph of page 18.

"Note that in both XSB and TBO, the latency cannot be zero in practice. We only displayed the result from VHLS C synthesis."

In the same paragraph, to address the FF problem, we have modified our analysis to the hardware resources of XSB as follows:

"XSB consumed only two FFs to perform multiplication, while TBO consumes 32 FFs. Each pair of FFs used to construct a logic gate. Therefore, XSB utilizes a logic gate, while TBO utilizes 16 logic gates."

Comment 8:

Because of lack of comparison with prior/related work and no discussion of prior/related work, the list of references is incomplete.

Reply 8:

We have added the discussion of prior works in the last paragraph of page 2. We have included the comparison with prior work, Rongshi et al [39]. in the Table 10 (page 20), 11 (page 21), and 12 (page 21).

Comment 9:

Fig. 1 is incorrect. The XNOR result of 1 and 1 is 1 and not 0.

Reply 9:

Thank you for mentioning this mistake. We corrected all "XNOR" to "XOR" throughout the paper.
