# Supplementary material for: Mixed-precision weights network for field-programmable gate array
Source: PLoS One. 2021 May 10;16(5):e0251329. doi: 10.1371/journal.pone.0251329 (PMC8109814; doi:10.1371/journal.pone.0251329)
Supplement: Attachment — Submitted filename: reply_letter.pdf [file pone.0251329.s002.pdf]

Revision Notes

(To be attached to a revised manuscript Reference No. PONE-D-20-36981R2)

April 6, 2021.

Dear Dr. Emily Chenette,
Editor-in-Chief,
PLOS ONE

Dear Reviewers,

Thank you very much for your kind consideration and careful evaluation of our following manuscript.

Reference No.: PONE-D-20-36981R2
Title: Mixed-precision weights network for field-programmable gate array implementation
Authors: Ninnart Fuengfusin and Hakaru Tamukoh

We have revised our paper in line with the suggestions made by the associate editor and reviewers. We are extremely grateful for the thorough review and useful suggestions. We have incorporated these suggestions in the revised manuscript, which we feel has improved the quality and understandability of the paper.

We would like to inform that all number of page, reference, listing, and table in this letter referred to the Revised Manuscript with Track Changes.

We would like to resubmit our paper for publication in the PLOS ONE.
Thank you very much for your efforts and time.

Best regards,

Ninnart Fuengfusin
Kyushu Institute of Technology, Japan
fuengfusin.ninnart553@mail.kyutech.jp

Reply to reviewer 1:

Comment 1:

The authors have improved the manuscript from the previous submission and have addressed many of my earlier concerns. However, I remain skeptical about the novelty of the FPGA implementation, particularly the treatment regarding HLS. As some other reviewers have pointed out, the presented FPGA implementation of XSB and TBO are standard textbook techniques implementation of low bit width numerical operations. There is nothing wrong about these implementations, but at the same time I also do not see the novelty in their designs. As a result, the long discussion surrounding Listing 1 and Listing 2 in pages 9-10 seems fairly excessive to me and undermines the value of this paper.

Reply 1:

Thank you for your comments. We would like to address that even if our digital circuits, which are results from Listing 1 and 2, may not be novel designed circuits. One of our novelties in these listings realizes from how to bypass datatype constraints in Vivado High-level synthesis (VHLS). By converting *half* (half-precision floating-point) to *ap_int* (arbitrary-bit integer), we can access bitwise methods, and after we utilized all preferred bitwise operations, we can convert it back to *half* without any loss in information. This allows these digital circuits to be implement-able with VHLS and open a way to implements these circuits with a quantization neural network (QNN) with the floating-point activation and binary or ternary weights.

Comment 2:

I believe the authors may simply lay out the hardware design as part of the implementation details. To justify the novelty of the FPGA implementation, the authors should compare their implementations with other low-bitwidth (binary and ternary) DNN inference work, which is plentiful. The work of FINN, for example, allows binary inference network to be generated easily for FPGAs. There are many other similar frameworks for binary and ternary inference work on FPGA. The authors should compare their hardware implementation against them if they intend to claim novelty on their FPGA implementation. Otherwise, I believe the authors may simply focus on their novelty in deriving and utilising mixed precision implementations for inference. I think if the authors can demonstrate the benefit of mixed-precision network over standard fixed, but possibly low precision network.

Reply 2:

We have added a section to indicate our novelties comparing with FINN [24] and other QNNs that were implemented to FPGA from Page 3 in the last paragraph. In general, to the best of our knowledge, our FPGA implementation of MPWN is the first FPGA implementation that utilizes binary or ternary weights with floating-point activations. We also displayed that XSB and TBO can be used to replace the multiplication processes in binary and ternary layers, respectively. Even though we did not provide a comparison with FINN, we provided comparisons with another binarized neural network (BNN) [10] framework, GUINNESS [25], which we are more familiar as shown in Tables 10, 11, and 12.

We also included a comparison with BNN that contains both 1-bit weights and activations in Table 3. BNN promises a better alternative compared with MPWN in terms of hardware utilization. However, it performs

worse in terms of test accuracy comparing with our models.

Comment #3:

If the authors can demonstrate how FPGAs are good target platform for such mixed-precision network as opposed to CPUs or GPUs solution, then the value of the manuscript would be much higher.

Reply #3:

To address this point, we did a comparison between our FPGA implementation (Proposed directive) and a CPU ARM Cortex-53 on Table 8 on Page 19. Using TBO and FPGA parallelism, our FPGA implementation performs less latency than ARM Cortex-53 from 2.0 times to 11.77 times depend on weight layers.

Reply to reviewer 2

Comment #1:

Thank you for the review. The paper is much better.
As I could observe, the comments of the reviewers ahve been met.

Reply #1:

Thank you for your reviews. Your comments have improved this work.

Reply to reviewer 4

Comment #1

The authors have tried to address most of my comments. However, I am not convinced with the response to my comment number 1 (Reviewer 4). Points 1, 2 and 3 listed in the author response to this comment are contributions. But are they novel when compared to prior work, especially in computer architecture and FPGA journal or conferences? The proof of novelty is not there. For instance, why is ASB so important a consideration for the authors?

Reply #1:

Thank you for your comments. ASB is important to us because it is a metric that indicates the quality of our model and its ability to deploy into hardware devices. By defining this metric, we can utilize Bayesian optimization to automatically discover suitable combinations of weight layers to deploy into the FPGA.

We have added a section to indicate our novelties comparing with prior works in the FPGA field from Page 3 in the last paragraph. In general, to the best of our knowledge, our novelties in the FPGA field are we provide a first FPGA implementation of binary or ternary weights model with floating-point activation. To effectively deploy with binary or ternary weights and floating-point activations, we introduce XSB and TBO to replace floating-point multiplications with bitwise operations instead.

Comment #2

The response to my comment 6 says that hardware results are compared with Rongshi et al [39]. But is that work the state-of-the-art work to make comparison with since comparison is being done only with one work?

Reply #2:

The main reason that we have compared our work with Rongshi et al [26] is Rongshi et al. provides the same architecture (LeNet-5) with our work to compare with. For the same reason, we have added Cho et al [27] and GUINNESS [25] to compare with as shown in Tables 10, 11, and 12. Our model performs with less latency comparing than the prior works with a cost that our work consumes more hardware utilizations.

Comment #3

The response to my comment 7 says "Each pair of FFs used to construct a logic gate". That is a fundamentally incorrect statement in digital logic and design. FFs cannnot be used to construct a logic gate. In fact, logic gates can be used to construct flip flops (FFs).

Reply #3:

Thank you for mention this issue. We have checked the results and found out that we did typos from LUT to FF. We have adjusted our statement from “Each pair of FFs used to construct a logic gate.” to "Each pair of LUTs used to construct a logic gate." instead. We have corrected these mistakes throughout the paper.
